# AP-PA field orientation followed by IMRT reduces lung exposure in comparison to conventional 3D conformal and sole IMRT in centrally located lung tumors

**DOI:** 10.1186/1748-717X-7-23

**Published:** 2012-02-16

**Authors:** Viacheslav Soyfer, Yaron Meir, Benjamin W Corn, Dan Schifter, Eliahu Gez, Haim Tempelhoff, Natan Shtraus

**Affiliations:** 1 Tel Aviv Sourasky Medical Center, Radiation Oncology Department, 6 Weizman Street, Tel Aviv 64239, Israel

**Keywords:** Lung cancer, Radiation, IMRT, 3D, AP-PA

## Abstract

Little attention has been paid to the fact that intensity modulated radiation therapy (IMRT) techniques do not easily enable treatment with opposed beams. Three treatment plans (3 D conformal, IMRT, and combined (anterior-posterior-posterio-anterior (AP-PA) + IMRT) of 7 patients with centrally-located lung cancer were compared for exposure of lung, spinal cord and esophagus. Combined IMRT and AP-PA techniques offer better lung tissue sparing compared to plans predicated solely on IMRT for centrally-located lung tumors.

## Background

Lung cancer constitutes a major source of mortality in the western world. The disease is usually diagnosed in advanced stages. Locally advanced disease represents 22% of newly diagnosed cases and is typically associated with 5 year relative survival rates of 24% [1]. External beam radiation therapy is the essential component in the treatment of stage 3 Non Small Cell Lung Cancer (NSCLC) and Small Cell Lung Cancer (SCLC) patients. Definitive radiation concurrent with chemotherapy is the standard of care of stage 3 NSCLC. Recommended doses of radiation therapy usually are in the range of 60 to 70 Gy [2,3]. Wang et al. demonstrated the importance of dose escalation on overall survival in stage 3 NSCLC patients [4]. The impact of radiation therapy on local control and survival in SCLC has also been described [5,6]. The recommended dose schedules for radiation treatment of SCLC are either 1.5 Gy twice daily to 45 Gy total dose or 2 Gy once daily to 60-70 Gy according to NCCN Guidelines (Version 2.2012) [7]

In delivering radiation therapy to the lung and mediastinum, attention must be devoted to tolerance of normal tissues. The lung itself is regarded as a very sensitive organ to radiation damage. Many publications have addressed the consequences of radiation pneumonitis, which might be a life threatening complication, and depends on the dose of radiation [8]. The tolerance of the esophagus and spinal cord to radiation is not less important. Singh et al. established the threshold point dose for Grade 3-5 esophageal toxicity at 58 Gy [9]. Published studies have reported a wide range of parameters to establish the probability of esophageal radiation related toxicity. Lievens et al. believe that maximal doses rather than mean doses are more determinant of late toxicity such as stricture and ulceration [10]. Dose-volume data for myelopathy in humans treated with radiotherapy to the spine was reviewed by Kirkpatrick et al. Using conventional fractionation of 1.8-2 Gy/fraction to the full-thickness cord, the estimated risk of myelopathy is < 1% and < 10% at 54 Gy and 61 Gy, respectively, with a calculated strong dependence on dose/fraction (alpha/beta = 0.87 Gy.) [11].

Given the above considerations, IMRT techniques were adopted by some centers in the treatment of lung cancer patients. Comparative evaluation of this technique with commonly used 3D conformal treatment showed a preference in terms of the target coverage and the lung exposure and thereby the probability of radiation pneumonitis. Liu et al. advocated reduction of V10 and V20 for thoracic normal tissues using IMRT. The percentage of lung volume that received > 20 Gy and the mean lung dose were reduced for all cases with reduction for a median of 8% and 2 Gy respectively [12]. Murshed et al. described the median absolute reduction of lung volume irradiated to > 10 and 20 Gy using IMRT as 7% and 10% respectively. This corresponds to a decrease of > 2 Gy in the total lung mean dose and of 10% in the risk of radiation pneumonitis [13].

The benefit of single IMRT approaches to treat thoracic malignancies was questioned by several authors. The high incidence of fatal pneumonitis after IMRT irradiation for mesothelioma was attributed to large volumes of lung receiving

low doses (V5 > 90%) [14]. Mayo et al. introduced the notion of hybrid IMRT for treatment of lung and esophageal cancer. They compared concurrently combined static and IMRT beams with 3D and IMRT plans. Hybrid plans resulted in smaller total and contra lateral lung volumes with lower doses than IMRT plans [15].

The new technology of volumetric arc therapy (VMAT) was also compared with Hybrid- RapidArc treatment for NSCLC and showed the lowest V20 and MLDs [16]

We propose that the delivery of radiation therapy with AP-PA beams followed by IMRT may actually improve the sparing of normal lung tissue while possibly increasing doses to the mediastinal structures, yet still maintaining the latter within the range of tolerability. The hypothesis is based on the fact that with IMRT field arrangements, opposed beams are not recommended. Indeed Bortfeld, on behalf of the American Association of Physicists in Medicine (AAPM, Monograph 29), admonished that "parallel-opposed beams should be avoided in IMRT - the reason (being) that parallel opposed beams add much less beam-shaping potential" [17].

With "inverse planning" which is embedded in IMRT techniques the user sets the beams angle arrangements, and the optimization engine determines the best solution for the leaf arrangement with corresponding dose per beam.

Using opposed fields will cause the optimization engine to be less effective because of the debate regarding which beam will deliver the dose to the target.

Therefore, theoretically, in centrally located lung tumors more intact lung tissue is irradiated and organs of the mediastinum are relatively spared in the IMRT technique. We therefore suggest that older AP-PA techniques not be indiscriminately abandoned for all patients undergoing definitive irradiation following a diagnosis of lung cancer.

The combination of AP-PA with the aim of sparing the lung tissue and IMRT in an attempt to preserve the esophagus and spinal cord might be considered for treatment planning.

## Methods

Seven patients with centrally-located primary lung cancer were assessed for the treatment plans described above in our department. Four of them had NSCLC and three- Limited Stage SCLC. There were four men and three women, age 43 to 70 year old. (mean: 67). The dose prescribed to the PTV was 60 Gy in fractions of 2 Gy.

PET CT was performed on every patient prior to simulation. All patients underwent CT simulation; 3 mm slice thickness (Bigbore 120 - Brilliance, Phillps) in the supine position on the lung board. The instructions for the PTV delineation were strictly followed in accordance with the ICRU- 62 guidelines [18]. The GTV included the visible tumor based on the pretreatment PET CT appearance. Lymph nodes were electively irradiated in cases of SCLC. Gross tumor volume (GTV)-to clinical target volume (CTV) margins of 5 mm were applied. Margins of 5 mm to 10 mm depending on tumor location and the vicinity of critical structures were added to generate the planning target volume (PTV) primarily in order to account for target motion [18-20]. Daily cone beam CT guidance was used in order to minimize set-up errors and to reduce the need to incorporate larger margins into the PTV.

Organs at risk (OAR) - heart, spinal cord, esophagus and lungs were delineated by the treating physician. The spinal cord was delineated for its length in the treatment field. The outer wall of esophagus was contoured in the length of the treatment field as well. The heart was delineated from the apex to the base and the lungs were automatically contoured using the Hounsfield unit threshold algorithm.

Three plans were subsequently generated using consistent planning parameters such as field arrangements, optimization parameters and beam weights. First, a 3D plan using 7-9 conformal beams (XiO, v 4.4 CMS). Second, a strict IMRT plan using 7 beams. Third, a plan that consisted of two sequentially administered techniques: IMRT and AP-PA ("experimental plan"). The dose calculations were performed using homogeneity corrections and superposition algorithm. Physicians selected the preferred plan after comparing dose distributions by carefully assessing the best DVHs with attention to V5, V10, V20, V30 and V40. Mean dose of the lung DVH, maximal dose to the esophagus and spinal cord were also studied. The most frequently selected energy of photons was 10 MV. The number of the fields per planning was seven to nine for the conformal 3D, seven for IMRT and for the combined technique- seven for IMRT plus separately prescribed AP-PA fields.

The weighting of the opposed fields against IMRT in the combined group was equally distributed.

The OAR limitations were as follow: MLD (mean lung dose) less than 19 Gy, V20- less than 37%, heart (V heart 50- less than 67%, V heart 60- less than 33%), esophagus D max - less than 66 Gy, spinal cord- D max - less than 50 Gy). PTV coverage should be at 95% of the planned dose and the maximal dose less than 107%.

Inhomogeneity corrections were applied to all cases during the planning process. A super-position calculation algorithm was used for dose calculation. Daily set up error correction was managed by the cone beam CT and laser-assisted patient positioning on the lung board.

The statistical approach was to extract the two-tailed P-Value via independent T testing, using two-sample distributions assuming unequal variances. Our hypothesis was that either IMRT or 3D is better than the combined technique and our alternative hypothesis was that the combined technique is superior.

## Results

**Figure 1:**
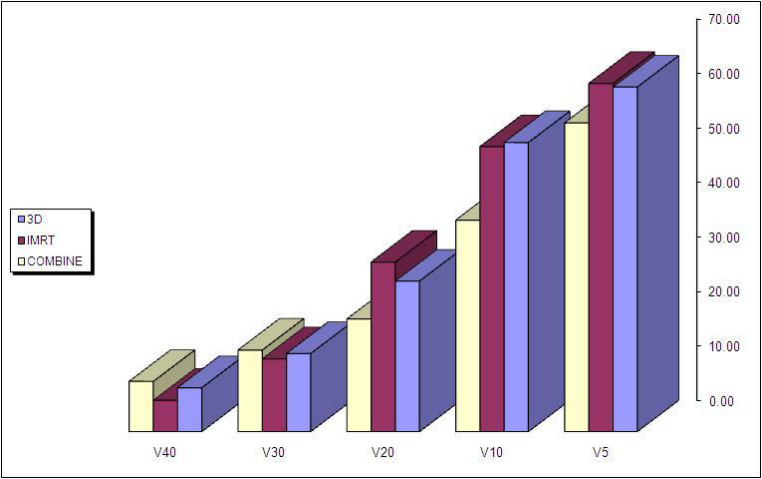
**Normalized DVH of PTV and Total Lung - GTV**. The graph depicts the relative dose- volume- histogram for the three techniques of 3D, IMRT and combined IMRT + AP-PA after the correction of heterogeneity

**Figure 2:**
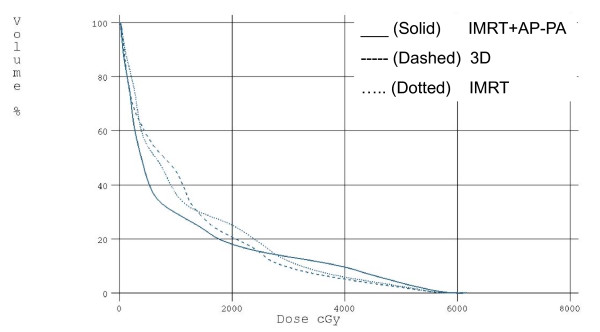
**Comparative axial plans**. The figure depicts the dose distribution of three techniques (3D, IMRT and combined IMRT + AP-PA) for a single representative patient

**Figure 3:**
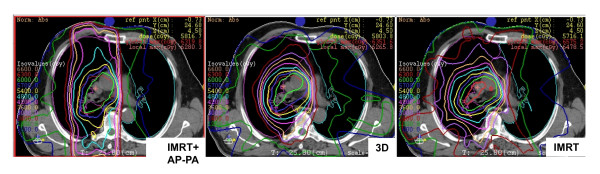
**Comparative DVHs of PTV and Total Lung - GTV**. The graph depicts the relative dose- volume- histogram for the three techniques of 3D, IMRT and combined IMRT + AP-PA

**Figure 4:**
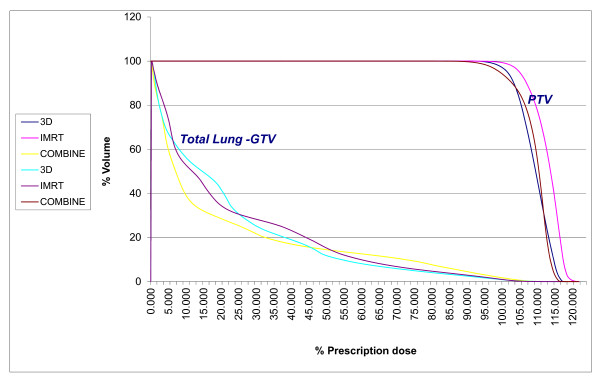
**Volumetric exposure of the total lung - GTV**. The figure depicts the comparative percentage of the total lung - GTV volume exposure related to the dose of radiation of 5 Gy, 10 Gy, 20 Gy and 30 Gy for three different techniques of the treatment (3D, IMRT and combined IMRT + AP-PA) grouped together for each dose superlatively

The PTV dose coverage with heterogeneity correction was equivalent in all three plans: 95.3 ± 4.9% (STDV), 95.5 ± 5.6%, 94.6 ± 2.3% in 3D, IMRT and combined (IMRT and AP-PA) plans relatively (Figure 1). The DVH of the lung in three comparative plans for 3D, IMRT and combined plans for the total lung -GTV was as follow: V5- 63.2 ± 8.6%, 63.9 ± 9.2%, 56.6 ± 11.4%; V10- 53.07 ± 10.1%, 52.3 ± 11.2%, 38.8 ± 9.6%; V20- 27.6 ± 6.7%, 31.1 ± 7.0%, 20.6 ± 2.3%; V30- 14.3 ± 4.6%, 13.36 ± 0.37%, 14.96 ± 1.22% and V40- 8.05 ± 4.62, 5.76 ± 2.15 and9.21 ± 1.62 respectively (Figure 2, 3). The mean dose to the right lung- GTV was 17.27 ± 5.47 Gy, 16.17 ± 4.59 Gy, 13.51 ± 5.42 Gy, respectively. The mean dose to the left lung - GTV was 12.1 ± 5.53 Gy, 13.27 ± 4.02 Gy, 9.31 ± 5.77 Gy. The maximal dose to the esophagus was 53.21 ± 3.05, 54.4 ± 4.67, 52.3 ± 4.5 Gy, respectively. Maximal dose to the spinal cord was 42.5 ± 2.9, 39.58 ± 1.2 and 43.7 ± 4.5 Gy respectively. DVHs and axial plans of the representative techniques are presented in Figure 3, 4

The number of monitor units was 527 ± 86.7, 669 ± 200.4, 670 ± 226.0 and 237 ± 32 for 3D, sole IMRT, IMRT and AP-PA parts of combined plan accordingly.

When examining the P-Value we found that there is a favored statistical outcome when using the combined technique vs. IMRT and 3D in the V10 and V20 region.

V10 and V20 were 0.025 and 0.019 respectively - when comparing IMRT and combined techniques, and 0.035 and 0.034 respectively - when comparing 3D and combined techniques. The difference between the combined technique and IMRT or 3D was not statistically significant in the V30 area (p = 0.96 and 0.413) and between combined and 3D in the V40 area (p = 0.706), whereas it *was *statistically significant between combined and IMRT in V 40 in favor of IMRT (p = 0.017) and in combined and 3D in V5 (p = 0.0001) in favor of combined, respectively. The differences in maximal doses to spinal cord and esophagus between the experimental (combined), IMRT and 3D were also not statistically significant:

P = 0.38 and 0.12 for 3D and IMRT for spinal cord and 0.14 and 0.238 for esophagus respectively. The data demonstrate that when using the combined technique over IMRT or 3D alone, there are better dosimetric results in terms of OAR.

## Discussion

Although used for decades, inherent challenges still exist in delivering radiation therapy to lung cancer patients. With prior techniques it was recommended to irradiate lung tumors with a two-step procedure beginning with AP-PA fields to 40 Gy, followed by coplanar fields in deference to spinal cord tolerance [21]. Three dimensional techniques significantly improved the dose delivery to the target volumes while maintaining normal tissues within acceptable dose limits. IMRT further optimized results as documented by several authors [12,13].

Arguably, the most important obstacle in achieving the maximal dose deposition for lung tumors is the lung itself. Indeed, radiation-related pneumonitis can constitute a fatal disease. In a seminal report, Graham et al. established V20 as the most important factor in the incidence of the pneumontis [7]. A V20 at 40% is associated with a 36% incidence of pneumonitis (> grade 2). In our hands, the V20 achieved with the combination of IMRT fields and AP-PA techniques was 21% in contrast to a V20 of 31% when IMRT was not supplemented by AP-PA portals. Other parameters such as V5, V10, and V30 have also been found to be determinant in the development of clinically significant pneumonitis [22]. V40 is rarely cited as a prognostic factor for radiation pneumonitis and is determined at 10% as the threshold level [23]. The results of the combined arm in V40 were inferior to IMRT, although they still were in the safe area below 10%.

A controversial issue that is emerging in the IMRT era is the implication of irradiating larger tissue volumes to lower doses. Shueng et al. [24] pointed out that sometimes a static set of AP-PA fields can be advantageous in view of the risks of large amounts of low dose irradiation to non-target organs at risk. Those authors did not endeavor to synthesize an AP-PA approach together with IMRT planning as was done herein. Shi et al. [25] have found that V10 above 50% is associated with a 29.2% risk of radiation pneumonitis. In contrast those authors saw a 5.7% risk of radiation pneumonitis when the V10 was maintained below 50%. Of interest, our experimental plan (i.e., the combination of AP-PA fields with IMRT techniques) reached a V10 value of 39% as opposed to a V10 of 53% when "pure" IMRT techniques were employed.

In our work we studied a clinical model in which the combination of IMRT and AP-PA techniques were compared to IMRT alone or to 3-D conformal technology alone. The theoretical rationale of such a combination exploits the sophisticated dose shaping capabilities of IMRT coupled with the minimal dose contribution to normal lung derived as a consequence of AP-PA beam arrangement. Although the latter point may seem prosaic, we maintain that there is simply no substitute for the normal lung tissue avoidance achieved with rudimentary AP-PA techniques.

In our study we deliberately selected patients with centrally located tumors to emphasize the difficulties encountered in planning this common clinical presentation. In this clinical scenario the irradiation fields significantly involve the soft tissue of the mediastinum. Therefore, the strategy developed herein was to restore the use of the older technique of AP-PA beam arrangements in the combined treatment plans in order to avoid excess tracking of radiation through the lung tissue.

## Conclusions

Our results show that the use of AP-PA field orientation followed by IMRT offers benefit in terms of critical lung volume irradiation while maintaining the esophagus and the spinal cord within tolerable limits.

## Abbreviations

IMRT: Intensity modified radiation therapy; AP-PAm: Anterio-posterior-posterio-anterior; NSCLC: Non Small Cell Lung Cancer; SCLC: Small Cell Lung Cancer; GTV: Gross Tumor Volume; PTV: Planning Target Volume; OAR: Organs at risk; MLD: Mean lung dose

## Competing interests

The authors declare that they have no competing interests.

## Authors' contributions

VS, NS and YM conceived the study, collected data and drafted the manuscript. BWC, DS and EG participated in coordination and helped to draft the manuscript. VS and BWC provided the mentorship and edited the manuscript. All authors have read and approved the final manuscript.
